# Development and Psychometric Validation of a Questionnaire Assessing Gynecologic Oncology Nurses’ Knowledge, Attitude, and Practice in Providing Sexual Health Care

**DOI:** 10.3390/healthcare14142049

**Published:** 2026-07-08

**Authors:** Yanxia Sun, Jianchen Zhang, Baoxin Shi

**Affiliations:** 1 School of Basic Medical Sciences, Tianjin Medical University, 22 Qixiangtai Road, Heping District, Tianjin 300070, China; syx1106@126.com (Y.S.); zhang_jian_chen@126.com (J.Z.); 2 Hospice Research Center, Tianjin Medical University, 22 Qixiangtai Road, Heping District, Tianjin 300070, China

**Keywords:** gynecologic oncology, sexual health care, questionnaire development, reliability, validity

## Abstract

**Background/Objectives**: The objective of this study was to develop a questionnaire assessing knowledge, attitudes, and practices regarding sexual health care among gynecologic oncology nurses and evaluate its psychometric properties. **Methods**: The questionnaire was developed using the Knowledge, Attitude, and Practice (KAP) framework as its theoretical foundation, drawing on an extensive literature review and qualitative interviews. The development process was further refined through two rounds of Delphi expert consultation and pilot testing. Using convenience sampling, 517 nurses working in gynecologic oncology settings were recruited from nine hospitals across six provincial-level regions in China (Tianjin, Shanghai, Hebei, Xinjiang, Hunan, and Hainan) between November and December 2024. Participants were randomly split into two distinct subsamples, with one allocated to exploratory factor analysis and the other to confirmatory factor analysis. **Results**: The final version of the questionnaire included 40 items covering three dimensions. It yielded an overall Cronbach’s α of 0.980, a split-half reliability of 0.919, and a test–retest reliability of 0.982, with the mean content validity index being 0.956. Subsequent exploratory factor analysis yielded three common factors, with item loadings spanning 0.539 to 0.809, and the cumulative variance explained reached 70.467%. The confirmatory factor analysis yielded an adequate model fit, which confirmed the structural stability of the questionnaire. **Conclusions**: The questionnaire exhibited high reliability and validity, thus making it a rigorous and dependable instrument for evaluating the knowledge, attitudes, and practices of gynecologic oncology nurses in regard to sexual health care.

## 1. Introduction

The global burden of gynecologic malignancies has been rising steadily over the past several decades. In 2022, 1.47 million women worldwide were newly diagnosed with gynecologic cancers. With continuous advances in early cancer screening, precision diagnosis and treatment, and rehabilitation support systems, the survival of patients with gynecologic malignancies has improved markedly, with the 5-year survival rates for cervical, endometrial, and ovarian cancer reaching 62%, 81%, and 40%, respectively [1]. Owing to cancer location, the extent of surgery, the damage caused by radiotherapy and chemotherapy, and changes in hormone levels, sexual dysfunction is one of the common adverse clinical outcomes following treatment for gynecologic malignancies, with an incidence rate as high as 93.7%, which is substantially higher than that among patients with other types of cancer [2]. It seriously impairs patients’ sexual and overall health. Increasing attention has been paid by the medical community and society to patients’ quality of life and mental and sexual health [3]. Sexual health is a state of physical, emotional, mental, and social well-being in relation to sexuality. As a key dimension of overall health and rehabilitation, sexual health directly affects family functioning, social adaptation, and long-term prognosis [4,5]. Most patients with gynecologic cancers have sexual health care needs, and health care professionals should proactively assess these needs, provide scientific guidance, and deliver standardized sexual health support [5,6].

As integral participants of the oncology care team, nurses serve as frontline providers in sexual health assessment, patient–provider communication and health education, rehabilitation interventions, and interdisciplinary care linkage [7,8,9]. Sexual health care involves not only targeted rehabilitation nursing for patients who have developed sexual dysfunction but also proactive preventive health education for cancer patients at high risk of sexual impairment [10]. Oncology nurses’ knowledge base, attitudes, and practical competence in sexual health nursing directly determine the quality and accessibility of sexual health care in clinical practice [11]. The Knowledge, Attitude, and Practice (KAP) theoretical framework notes that knowledge forms the foundation for behavioral change, attitude functions as the intrinsic impetus behind behavioral transformation, and practice is the ultimate goal of translating knowledge and beliefs into action [12]. However, problems such as insufficient knowledge, conservative attitudes, and inadequate practice in sexual health nursing are widespread among gynecologic oncology nurses, and there remains a lack of unified, scientific, and highly targeted evaluation instruments, thus making it difficult to comprehensively evaluate their knowledge, attitudes, and practice regarding sexual health care [13]. Therefore, guided by the KAP theoretical framework and using a combination of a systematic literature review, semi-structured interviews, and the Delphi expert consultation method, the present study developed a questionnaire for assessing gynecologic oncology nurses’ knowledge, attitude, and practice in providing sexual health care and evaluated its reliability and validity. This study aimed to establish a rigorous and dependable instrument for the scientific evaluation of gynecologic oncology nurses’ sexual health care competence, the targeted implementation of specialist training, and the promotion of standardized clinical sexual health care.

## 2. Methods

### 2.1. Establishment of Study Group

The study group comprised two master’s supervisors in nursing, one chief nurse, one associate chief nurse, two postgraduate nursing students, and two clinical nurses. The team’s core tasks involved carrying out a literature review; conducting semi-structured interviews; developing, distributing, and retrieving expert consultation questionnaires; integrating expert feedback; and performing data analysis and statistical processing.

### 2.2. Construction of Questionnaire Item Pool

#### 2.2.1. Theoretical Basis

The KAP (Knowledge, Attitude, and Practice) framework maintains that knowledge provides the necessary foundation, constructive attitudes and beliefs act as internal drivers, and the adoption and evolution of practice behaviors mark the final objective [10]. Based on this theoretical framework, the research team organized the questionnaire into three dimensions: knowledge, attitude, and practice. The KAP assessment tool developed in this study adopted a 5-point Likert self-rating questionnaire to evaluate nurses’ subjective perceptions of their own levels of knowledge, attitudes, and practices related to sexual health care. Compared with objective standardized written examinations and practical skill assessments, this tool is time- and labor-saving and can better reflect nurses’ willingness in terms of clinical care and their actual nursing practices. (1) Knowledge: This dimension assessed gynecologic oncology nurses’ understanding of basic theoretical knowledge related to sexual health, manifestations of sexual dysfunction, and factors influencing sexual health in cancer patients. (2) Attitude: This dimension assessed attitudes toward both sexual health issues among cancer patients and the provision of sexual health care and self-perceived role awareness in sexual health care practice. (3) Practice: This dimension assessed gynecologic oncology nurses’ competence in the assessment and management of and nursing practice related to sexual health issues in cancer patients.

#### 2.2.2. Literature Review

The search terms included “nurse/health caregiver”, “cancer*/tumor*/neoplasms”, and “sexuality/sexual dysfunction/sexual adjustment/sexual life/sexual behavior/sexual problem”. A systematic search was conducted in the following databases: PubMed, Embase, The Cochrane Library, Web of Science, China National Knowledge Infrastructure, Wanfang Data, VIP Database, and SinoMed. In addition, the reference lists of included studies were manually screened for supplementary retrieval. The search period covered database inception to August 2024. Based on the KAP theoretical model, the included literature was reviewed and analyzed to construct the initial structural framework and item pool of the questionnaire. The preliminary questionnaire comprised three dimensions—knowledge (12 items), attitude (13 items), and practice (17 items)—with a total of 42 items.

#### 2.2.3. Semi-Structured Interviews

We adopted purposive sampling to recruit 12 nursing professionals from a tertiary A-level oncology-specialized hospital in Tianjin and conducted semi-structured interviews with them in September 2024. Participants satisfying the following criteria were included: (1) possessing a current registered nurse license; (2) being a gynecologic oncology nurse or nurse with experience in caring for patients with gynecologic malignancies; (3) having accumulated at least 1 year of work experience in the field; and (4) agreeing to participate in this research. Individuals were excluded if they met the following criteria: (1) nurses currently in training programs or internships or (2) staff who were absent from work due to sick leave.

The interview guide included the following questions: (1) When providing sexual health-related care services for patients diagnosed with gynecologic cancers, what knowledge and theories do you consider essential to understand and master? (2) What are your attitudes, views, feelings, and experiences regarding sexual health issues and sexual health care provided to patients with gynecologic malignancies? (3) What do you consider to be the basic content and core competencies required for sexual health care provided to patients with gynecologic malignancies? (4) What suggestions do you have regarding sexual health care practice?

Drawing on the literature review and qualitative interview results, the research group iteratively refined and revised the measurement tool. The initial version of the questionnaire included three core dimensions, knowledge (12 items), attitude (12 items), and practice (20 items), with a total of 44 items.

### 2.3. Delphi Expert Consultation

Purposive sampling was used to conduct Delphi expert consultation from September to October 2024. Experts satisfying the following criteria were included: (1) primary clinical or research experience in gynecologic oncology medicine, nursing, or sexual health medicine of no less than 10 years; (2) having obtained a bachelor’s degree or above; (3) holding an intermediate-level professional title or higher; (4) familiarity with the Delphi consultation process and questionnaire development procedures; and (5) agreeing to take part in this research.

The Delphi expert consultation questionnaire was structured into three sections: an instruction sheet, a form collecting experts’ basic information, and a consultation rating form. The instruction sheet included the research background, objectives, significance, and guidance on how to complete the questionnaire. The basic information form gathered details on demographic and professional characteristics, including age, educational level, and years of work experience, as well as experts’ self-rated familiarity (Cs) and judgment basis (Ca). The consultation form employed a 5-point Likert questionnaire for assessing the significance of all dimensions and items within the questionnaire.

Questionnaires were sent out and gathered through both email and on-site administration. The consultation process was terminated when expert opinions tended to converge, and the initial version of the Knowledge, Attitude, and Practice Questionnaire on Sexual Health Care for Gynecologic Oncology Nurses was then finalized. The selection criteria for items included a mean importance score > 3.5, a coefficient of variation < 0.35, and consensus criteria including ≥70% agreement [14].

### 2.4. Pilot Survey

Using convenience sampling, gynecologic oncology nursing staff from a tertiary A-level oncology-specialized hospital in Tianjin were recruited in October 2024 for a pilot survey. The criteria for participant inclusion and exclusion remained identical to those applied in the semi-structured interviews. By asking participants about their understanding of each questionnaire item and their response selection process, the wording of the questionnaire was further refined to improve clarity, readability, and accuracy.

### 2.5. Formal Survey

Using convenience sampling, gynecologic oncology nursing staff were recruited between November and December 2024 from nine hospitals across six provincial-level regions in China, including Tianjin, Shanghai, Hebei, Xinjiang, Hunan, and Hainan. Participants were divided into two independent groups, with one subset designated for exploratory factor analysis and the other for confirmatory factor analysis. Participant inclusion and exclusion criteria remained identical to those applied in the semi-structured interviews.

According to the principles of questionnaire development, the required sample size for exploratory factor analysis is 5 to 10 times the number of items, whereas for confirmatory factor analysis, the sample size should be larger than that used in exploratory factor analysis and exceed 200 [15]. The trial version of the questionnaire contained 42 items. Considering a projected 10% proportion of unusable responses, the sample size needed for this investigation was determined to be at least 462 individuals. We submitted the complete research protocol for ethical review prior to the commencement of the formal survey for the first time. The subsequent supplementary submission of relevant supporting documents led to the official ethical approval of this study by the Institutional Review Board of Tianjin Medical University Cancer Institute and Hospital (bc20250246) on 15 January 2025. This was not a deliberate post hoc approval procedure. All study procedures were strictly performed in accordance with the predefined protocol, and no practices that may impair the physical and mental rights and interests of participants were involved in this study.

The survey instruments comprised a general information collection form and the trial version of the Knowledge, Attitude, and Practice Questionnaire on Sexual Health Care for Gynecologic Oncology Nurses. The general information questionnaire collected data on sex, age, length of work experience, educational attainment, professional rank, position, whether the participant was a specialist nurse, and department. The pilot questionnaire employed a 5-point Likert questionnaire (1 = strongly disagree; 5 = strongly agree) for all items. Higher summed scores reflected better knowledge and more positive attitudes and practices regarding sexual health care.

With permission secured from the nursing managers of each hospital, trained members of the research team distributed the questionnaire link to participants via an online survey platform. The opening section detailed the research goals. The opening instruction clearly stated that all answers were for research only, no right or wrong answers existed, and all data would be stored confidentially, and questionnaires were completed anonymously without collecting identifiable personal information. Guidance on how to fill out the survey was also offered. The questionnaire could only be submitted after every item had been answered. After the submission deadline, the data were exported in a timely manner and cross-checked independently by two researchers. Questionnaires completed in less than 1 min or showing obvious response patterns or logical inconsistencies were excluded.

#### Reliability and Validity Testing of Questionnaire

Item analysis (1) Critical ratio method: Questionnaire responses were sorted in descending order based on their total scores. The highest- and lowest-scoring 27% were designated as the high- and low-score groups, respectively. Differences between the two groups were evaluated via an independent-samples *t* test. Only items that met the criteria of a critical ratio ≥ 3 or a significance level of *p* ≤ 0.05 were retained in the questionnaire. (2) Correlation coefficient method: Pearson correlation coefficients were computed between each item and the overall questionnaire score. Items that met the criteria of a correlation coefficient > 0.4 or a *p* value ≤ 0.05 were included in the final questionnaire. An inter-item correlation matrix within each dimension was further created to judge item redundancy. (3) Cronbach’s α coefficient method: Items were assessed based on their impact on the questionnaire’s reliability. Any item that caused the overall Cronbach’s α value to rise when deleted was removed from the questionnaire [16].

Validity testing (1) Content validity: Content validity was evaluated by calculating the item-level content validity index (I-CVI) and scale-level content validity index (S-CVI). An I-CVI ≥ 0.78 and S-CVI ≥ 0.80 were deemed to demonstrate adequate content validity. (2) Construct validity: Exploratory factor analysis based on maximum likelihood extraction was adopted, with oblique Promax rotation selected as the primary analytical method (this rotation is theoretically preferred for correlated KAP constructs). The criteria were as follows: an eigenvalue > 1, cumulative variance explained > 50%, and a factor loading > 0.40 for each item on a single factor. Confirmatory factor analysis was performed using the maximum likelihood method. The evaluation criteria were standardized factor loadings > 0.45; chi-square/degrees of freedom (χ^2^/df) < 3; root mean square error of approximation with 90% confidence interval (RMSEA) < 0.08; Standardized Root Mean Square Residual (SRMR) < 0.06; and goodness-of-fit index (GFI), comparative fit index (CFI), incremental fit index (IFI), and Tucker–Lewis Index (TLI) all > 0.90, indicating an acceptable model fit. (3) Convergent validity: Convergent validity was considered acceptable when the average variance extracted (AVE) was >0.50 and composite reliability (CR) was >0.70. (4) Discriminant validity: Discriminant validity was considered acceptable when the square root of the AVE for each factor exceeded the correlation coefficient between that factor and any other factor and the heterotrait–monotrait ratio (HTMT) was <0.90 [16].

Reliability testing (1) Internal consistency: Cronbach’s α coefficients were calculated for the whole scale and each dimension. The questionnaire was judged to have good internal consistency if the Cronbach’s α coefficients of each dimension and the overall scale were no less than 0.70 and the deletion of any individual item did not lead to a substantial increase in Cronbach’s α. Meanwhile, corrected item–total and inter-item correlations were reported to assess item redundancy. (2) Split-half reliability: Split-half reliability for each dimension and the overall questionnaire was calculated using the odd–even split method. (3) Test–retest reliability: A sample of 50 nurses working in gynecologic oncology units was randomly selected to retake the questionnaire after a 2-week period. Test–retest reliability was deemed satisfactory if there was a correlation coefficient > 0.70 [16].

### 2.6. Statistical Analysis

All data were double-entered and analyzed using SPSS version 27.0 and AMOS version 26.0. Categorical data were presented as frequencies and percentages. Continuous variables following a normal distribution were reported as mean ± standard deviation, whereas those with a non-normal distribution were expressed using the median and interquartile range. The degree of expert participation was calculated from the effective questionnaire response rate. The authority coefficient (Cr) served as the measure for evaluating expert authority, variability was described by the coefficient of variation, and the degree of coordination was assessed using Kendall’s coefficient of concordance. A two-sided *p* < 0.05 was considered statistically significant.

## 3. Results

### 3.1. Results of Delphi Expert Consultation

The Delphi consultation was conducted in two rounds. For the first round, questionnaires were sent to 16 experts, with 15 valid responses returned (93.75%). The second round achieved full participation, with a valid response rate of 100%.

The 15 experts were enrolled from tertiary grade A general hospitals, specialized hospitals, and medical universities in multiple provinces and municipalities, including Beijing, Tianjin, Hebei, and Hainan. Their average age was 42.50 ± 2.00 years, with a mean of 17.50 ± 1.80 years of professional experience. Among them, five were engaged in gynecologic oncology medicine and nine in gynecologic oncology nursing, and one was a specialist in sexual medicine. In terms of educational level, six, six, and three possessed doctoral, master’s, and bachelor’s degrees, respectively. Regarding professional title, 5 held senior professional titles and 10 associate senior-level titles. Across the two consultation rounds, the expert authority coefficients were 0.862 and 0.897. The coefficients of variation for the items ranged from 0 to 0.211 in the first round and 0 to 0.196 in the second. The Kendall’s W values for the two rounds were 0.261 and 0.314, both reaching statistical significance (*p* < 0.001).

Using the item selection criteria and input from experts, the questionnaire items were revised. Specifically, after the first round of expert consultation, two items were removed, three new items were introduced, six items were combined, and the phrasing of eleven items was adjusted. Following the second round, no items were removed or added, while four items underwent wording refinements. After these two rounds of expert consultation, the initial questionnaire comprised three dimensions—knowledge (12 items), attitude (10 items), and practice (20 items)—with a total of 42 items.

### 3.2. Results of Pilot Survey

In the pilot survey, 20 questionnaires were sent out. The 20 pilot participants covered nurses with diverse ages, working years, and professional titles, which meets the standard sample size requirement for cognitive item comprehension testing in scale development research. Each participant completed the questionnaire in 5–10 min and indicated no difficulty in reading or understanding the items. Consequently, no items were removed, added, or revised.

### 3.3. General Characteristics of Participants

A total of 530 questionnaires were sent out, and 512 valid ones were retrieved, corresponding to a valid return rate of 96.60%. The valid questionnaires were then randomly split into two subsamples, 245 for item and exploratory factor analyses and 267 for confirmatory factor analysis.

The ages of the 512 participants spanned from 24 to 54 years old, with an average of 35.35 ± 7.10 years. Regarding educational background, 67 participants (13.09%) held a junior college diploma, 442 (86.33%) possessed a bachelor’s degree, and 3 (0.59%) had a master’s degree or higher. As for professional title, 75 (14.65%) were nurses, 256 (50.00%) were senior nurses, 169 (33.01%) were nurses-in-charge, 7 (1.37%) were associate chief nurses, and 5 (0.98%) were chief nurses. A total of 103 participants (20.12%) were oncology specialist nurses. Their work experience spanned 2 to 34 years, averaging 12.09 ± 7.75 years.

### 3.4. Item Analysis

The item analysis results were as follows: (1) Critical ratio method: Across the 245 questionnaires, the differences between the high- and low-scoring groups for all items were statistically significant. The t values for all items were >3, and all *p* values were <0.01. Consequently, none of the items were deleted. (2) Correlation coefficient method: The correlation coefficients for all items were >0.4, and all *p* values were <0.01. (3) Internal consistency coefficient method: After deleting each item one by one, the overall Cronbach’s α coefficient did not increase substantially. Taken together, all 42 items were retained.

### 3.5. Validity Testing

#### 3.5.1. Content Validity

The item-level content validity index (I-CVI) fell between 0.889 and 1.00, while the scale-level content validity index (S-CVI) was 0.956, reflecting satisfactory content validity for the questionnaire.

#### 3.5.2. Construct Validity

Exploratory factor analysis: The results of the first exploratory factor analysis showed that the Kaiser–Meyer–Olkin (KMO) value was 0.867, and Bartlett’s test of sphericity produced a χ^2^ value of 5268.144 (*p* < 0.001), thus suggesting that the data were suitable for factor analysis. By employing maximum likelihood extraction with oblique Promax rotation, four common factors with eigenvalues exceeding 1 emerged, cumulatively accounting for 68.251% of the variance. The fourth factor extracted only contained two cross-loading items without independent thematic connotation, thus lacking stable theoretical support and interpretability (item 1: “masters the important impact of sexual health on health outcomes among cancer patients and their spouses”; item 2: “master appropriate sexual health education models for cancer patients”), and these items were therefore deleted.

In the second exploratory factor analysis, the KMO value was 0.926, and Bartlett’s test of sphericity produced a χ^2^ value of 5960.022 (*p* < 0.001). Three common factors with eigenvalues > 1 were extracted, with a cumulative variance explained of 70.467%. Factor loadings for the retained items ranged from 0.539 to 0.809, and no cross-loading was observed; therefore, no further items were deleted. The extracted common factors were consistent with the predefined dimensions. The three factors were named knowledge (items 1–10), attitude (items 11–20), and practice (items 21–40), comprising a total of 40 items (Table 1).

Confirmatory factor analysis: The results showed that the model had a χ^2^/df of 2.538, root mean square error of approximation (RMSEA) of 0.067 (90%CI [0.052, 0.081]), Standardized Root Mean Square Residual (SRMR) of 0.053, goodness-of-fit index (GFI) of 0.917, comparative fit index (CFI) of 0.956, incremental fit index (IFI) of 0.926, and Tucker–Lewis Index (TLI) of 0.934. These findings suggested a satisfactory model fit and robust factor structure for the questionnaire. During confirmatory factor analysis, no correlated residual paths were added based on modification indices during modeling.

Criterion-related validity: No external mature specialized scale for gynecologic oncology nurses’ sexual health KAP was available for criterion comparison in the current stage; this limitation was fully acknowledged, and a future verification plan with behavioral criteria is proposed in the Discussion.

### 3.6. Convergent Validity and Discriminant Validity

The average variance extracted (AVE) values for the three dimensions were 0.832, 0.573, and 0.653, and the composite reliability (CR) values were 0.972, 0.930, and 0.974, respectively, thus indicating the satisfactory convergent validity of the questionnaire. In addition, the square root of the AVE for each dimension was greater than the correlations between factors (Table 2) and the heterotrait–monotrait ratio (HTMT) was <0.90 (Table 3), thus suggesting that the items in each dimension accurately reflected the construct intended to be measured. These results demonstrated the good discriminant validity of the questionnaire.

### 3.7. Reliability Testing

The overall Cronbach’s α coefficient of the questionnaire was 0.980, corrected item–total correlations were 0.414~0.896, and inter-item correlations were 0.218~0.890. The Cronbach’s α coefficients for the three dimensions were 0.970, 0.927, and 0.973. The overall split-half reliability coefficient was 0.919, and those for the three dimensions were 0.981, 0.935, and 0.955. Moreover, the overall test–retest reliability coefficient was 0.982, and those for the three dimensions were 0.976, 0.923, and 0.975.

## 4. Discussion

In the process of developing the questionnaire, the research team performed a thorough literature review to systematically identify relevant domestic and international evidence on sexual health care provided to patients with gynecologic malignancies, thereby ensuring that the questionnaire items covered core domains such as basic sexual health knowledge, attitudes toward care, and practical skills. In addition, semi-structured interviews with frontline nursing staff were conducted to thoroughly explore the needs and challenges encountered in clinical practice, thus allowing the questionnaire items to better reflect real-world nursing contexts. Furthermore, two consecutive Delphi rounds involving senior experts in gynecologic oncology medicine, nursing, and sexual medicine were conducted. The expert authority coefficients were greater than 0.85 for both rounds, and Kendall’s coefficients of concordance were statistically significant, thereby indicating good agreement among experts and further supporting the scientific rigor and professional relevance of the questionnaire content.

The reliability analysis revealed that the overall Cronbach’s α coefficient for the questionnaire was 0.980, and those for all dimensions exceeded 0.92, thus reflecting excellent internal consistency. The overall split-half reliability coefficient was 0.919, with dimension-level coefficients all being above 0.93, thereby suggesting the good stability of the questionnaire items. The overall test–retest reliability coefficient was 0.982, and those for all dimensions exceeded 0.92, thus indicating the strong reproducibility of the measurement results and minimal influence of time-related factors. While high reliability is desirable, the presence of such extremely high coefficients also implies potential item redundancy within dimensions. Although the analysis of corrected item–total and inter-item correlations showed no extremely duplicate items, the 40-item full version imposes a certain time burden on frontline nurses. In follow-up research, we will conduct systematic item reduction analysis based on corrected item–total correlations and factor loadings to develop a brief short-form questionnaire with comparable psychometric performance for rapid clinical screening. With respect to validity, the scale-level content validity index (S-CVI) was 0.956, and the item-level content validity indices (I-CVIs) ranged from 0.889 to 1.00, thus indicating that the questionnaire items were highly relevant to the study objectives and adequately reflected the core constructs of gynecologic oncology nurses’ knowledge, attitudes, and practices regarding sexual health care [17]. Exploratory factor analysis successfully extracted three common factors, which were fully consistent with the predefined dimensions of knowledge, attitude, and practice. The cumulative variance explained reached 70.467%, and item factor loadings ranged from 0.539 to 0.809 with no cross-loadings observed. Confirmatory factor analysis further showed that the model fit indices were acceptable, with χ^2^/df = 2.538, RMSEA = 0.067, and SRMR = 0.053, while the GFI, CFI, and other fit indices were all above 0.91, indicating that the questionnaire had a stable structure and good fit with the theoretical framework [18]. In addition, the average variance extracted (AVE) values for all dimensions were greater than 0.50, the composite reliability (CR) values were all above 0.93, and the heterotrait–monotrait ratio (HTMT) was <0.90. These findings indicated that the questionnaire had satisfactory convergent and discriminant validity and could effectively distinguish among different measurement domains, but the high correlation between knowledge and practice may indicate partial conceptual overlap between the two constructs. From a theoretical perspective, knowledge is the cognitive basis of behavioral practice; individuals with higher professional knowledge tend to implement more standardized practice behaviors, which naturally produces a strong correlation between the two dimensions. Future scale revision could delete several items with similar descriptions in these two dimensions to further reduce semantic overlap.

The questionnaire was structured around three dimensions—knowledge, attitude, and practice—and consisted of 40 items in total, featuring comprehensive content and a clear focus. The knowledge dimension covered core topics such as basic sexual health theory, the influence of cancer treatments on sexual function, and the assessment of sexual dysfunction, thus enabling a comprehensive evaluation of nurses’ professional knowledge reserves [19]. The attitude dimension focused on nurses’ perceptions of sexual health care, their acceptance of patients’ sexual health needs, and their self-perceived professional role, reflecting the intrinsic motivation to provide such care [20,21]. The practice dimension encompassed practical components such as sexual health assessment, communication skills, the implementation of interventions, and continuity of care, thereby comprehensively evaluating nurses’ clinical practice [22]. This survey instrument was tailored to the unique working characteristics of nurses in gynecologic oncology settings. Item phrasing was straightforward and comprehensible, the 5-point Likert questionnaire was easy to administer, and the questionnaire took only a short time to complete, thus making it appropriate for large-questionnaire survey studies [23,24,25]. In addition, the questionnaire has broad applicability and may be used to evaluate the knowledge, attitudes, and practices concerning the sexual health care provided by gynecologic oncology nurses with different professional ranks and years of work experience. It may also provide a scientific basis for health care institutions to develop targeted training programs and serve as a standardized measurement tool for related research, highlighting its important practical value.

In contrast to existing general oncology nursing tools, this questionnaire is uniquely tailored to the specific clinical context of gynecologic malignancies, covering sexual dysfunction mechanisms, treatment-related impacts, communication skills, rehabilitation guidance, and continuity of care—domains underrepresented in previous instruments [26,27,28,29]. Differences from international questionnaires may reflect cultural variations in attitudes toward sexual health, institutional care protocols, and specialty-specific nursing roles; the present tool was developed using domestic clinical evidence, frontline interviews, and multidisciplinary expert consensus to enhance contextual and cultural relevance. In clinical practice, the questionnaire supports the systematic identification of knowledge deficits, attitude barriers, and practice gaps, thus enabling nursing managers and educators to design targeted, evidence-based training programs; it also facilitates quality improvement, the standardization of sexual health care pathways, and enhanced survivorship care for women with gynecologic malignancies.

Despite these strengths, this study has identifiable limitations. These include the use of convenience sampling from nine hospitals across six provincial-level regions in China, which excluded primary, secondary, and rural medical institutions. Additionally, our sample contained a higher proportion of nurses with bachelor’s degrees and fewer nurses with junior college or master’s degrees (according to national statistics, the proportions of clinical nurses in China holding bachelor’s, junior college, and postgraduate degrees are 39.1%, 45.4%, and 0.5%, respectively), which may introduce selection bias and restrict generalizability to nurses from lower-level hospitals, smaller departments, or other geographic areas. The questionnaire employed a subjective self-assessment approach rather than objective evaluations or examinations, which may lead to the overestimation of nurses’ actual knowledge, attitudes, and competence. The questionnaire has not been tested for criterion-related validity or longitudinal sensitivity to training interventions. Cross-cultural or cross-linguistic validation has not yet been performed. Furthermore, cross-sectional self-reported questionnaire data inevitably suffer from common method bias and social desirability bias, particularly for sensitive topics related to sexual health. Although anonymous questionnaire completion was adopted to mitigate such biases, the confounding effects inherent to self-reported measurements cannot be fully eliminated. For future research, it is recommended to expand the sampling scope and adopt multi-stage stratified sampling to cover hospitals at all levels and rural medical institutions, enrolling clinical nurses with various educational backgrounds and professional titles, to reduce selection bias and improve representativeness. Longitudinal studies are needed to examine the questionnaire’s responsiveness to training and its predictive value for clinical performance. Researchers could adopt nurses’ cumulative training hours and real clinical sexual health counseling frequency as behavioral indicators to carry out criterion-related validity testing. Future studies may optimize the measurement method by combining objective knowledge tests and competency assessments. To reduce common method bias in future research, researchers should avoid collecting personal identifiable information, offer neutral instructions with no standard answers, randomize questionnaire items online, and apply Harman’s single-factor test in the Methods Section to detect this bias. Further investigation into factors influencing KAP levels will support precision intervention strategies. Additionally, this tool should undergo cross-cultural adaptation and validity verification through forward translation, back-translation, expert cultural adaptation evaluation, cognitive debriefing with local nurses, differential item functioning tests, and repeated psychometric testing among populations from diverse cultural backgrounds to expand its international applicability. Finally, the development of a short-form or digital version may improve usability in busy clinical settings.

## 5. Conclusions

The questionnaire assessing gynecologic oncology nurses’ knowledge, attitude, and practice in providing sexual health care demonstrates excellent reliability, high content validity, stable construct validity, and satisfactory convergent and discriminant validity. This instrument provides a rigorous, standardized tool for assessing nurses’ knowledge, attitudes, and practices related to sexual health care in gynecologic oncology settings.

## Figures and Tables

**Table 1:** The results of the exploratory factor analysis of the Questionnaire Assessing Gynecologic Oncology Nurses’ Knowledge, Attitude, and Practice in Providing Sexual Health Care (*n* = 245).

Item	Knowledge	Attitude	Practice
1. Has received or self-studied training or courses related to sexual health nursing	0.713	0.188	0.141
2. Understands the physiological and psychological characteristics of normal human sexual behavior	0.809	0.294	0.118
3. Understands the influence of demographic characteristics and sociocultural background on sexual needs and perceptions	0.752	0.288	0.093
4. Masters the influence of spouse-related factors (e.g., partner characteristics and relationship) on patients’ sexual function and psychology	0.607	0.285	0.271
5. Understands the importance of sexual health in the prognosis and intimate relationships of patients with gynecologic malignancies	0.787	0.287	0.284
6. Masters the effects of diagnosis and treatment of gynecologic malignancies on patients’ sexual function and psychology	0.764	0.266	0.175
7. Masters the clinical manifestations and assessment/diagnostic methods of sexual dysfunction	0.721	0.373	0.276
8. Is aware of the incidence of sexual dysfunction among patients with gynecologic malignancies	0.602	0.301	0.258
9. Masters scientific knowledge of sexual health nursing for patients with gynecologic malignancies	0.716	0.352	0.244
10. Masters the theories and applications of medical humanities care in sexual health nursing	0.638	0.317	0.167
11. Respects and understands patients’ different responses to sexual health topics (e.g., distress, fear, embarrassment, or expectation)	0.398	0.611	0.119
12. Respects and understands the sexual behaviors of special populations, including sexual minorities	0.163	0.682	0.132
13. Recognizes that nurses are important members in managing patients’ sexual health issues	0.255	0.774	0.133
14. Recognizes sexual health nursing as an important component of nursing work in the department	0.276	0.751	0.207
15. Expresses willingness to receive systematic training in sexual health nursing to conduct practice more scientifically	0.088	0.635	0.260
16. Is able to encourage patients and their spouses to consult about sexual health issues and proactively provide care	0.397	0.539	0.288
17. Does not feel embarrassed when patients or spouses initiate consultation and actively provides sexual health care	0.071	0.722	0.382
18. Is aware of personal strengths and limitations in sexual health nursing practice	0.332	0.791	0.209
19. Actively seeks solutions when unable to address patients’ sexual health problems	0.246	0.762	0.066
20. Believes that sexual health nursing does not cause adverse psychological or cognitive effects on oneself	0.334	0.717	0.153
21. The department has a systematic sexual health nursing program and/or a dedicated team	0.323	0.140	0.784
22. Is able to correctly handle various barriers and take full advantage of facilitating factors to carry out sexual health care services	0.341	0.185	0.686
23. Is able to clearly explain the effects of gynecologic malignancies and their treatments on sexual function	0.356	0.143	0.603
24. Is able to encourage patients to express their true feelings about sexual function changes	0.348	0.271	0.611
25. Is able to encourage communication between patients and spouses regarding sexual health issues	0.273	0.298	0.622
26. Is able to conduct individualized assessment of sexual health issues based on diagnosis, treatment, and complaints	0.333	0.157	0.732
27. Is able to use professional and accessible language to discuss sexual health topics with patients	0.263	0.384	0.606
28. Is able to discuss sexual health topics with patients at appropriate stages of treatment	0.375	0.236	0.632
29. Is able to discuss sexual health topics with patients in appropriate situations	0.281	0.223	0.671
30. Is able to select appropriate content to discuss sexual health topics with patients	0.246	0.327	0.722
31. Is able to use appropriate communication methods or health education models to discuss sexual health topics with patients	0.371	0.285	0.622
32. Is able to help patients and their spouses cope appropriately with cancer-related changes in sexual function	0.277	0.212	0.724
33. Is able to provide individualized recommendations on resuming sexual activity based on diagnosis and treatment	0.283	0.361	0.615
34. Is able to provide individualized guidance on sexual rehabilitation (e.g., pelvic floor exercises)	0.313	0.247	0.604
35. Is able to provide psychological interventions (e.g., cognitive therapy, meditation) to promote recovery of sexual activity	0.193	0.226	0.799
36. Is able to instruct patients on the use of assistive devices (e.g., lubricants)	0.328	0.273	0.621
37. Is able to guide patients and spouses in alternative ways to maintain intimacy	0.380	0.297	0.618
38. Is able to provide contraception and fertility guidance for patients wishing to preserve fertility	0.385	0.343	0.618
39. Is able to provide continuous sexual health nursing after patient discharge	0.292	0.249	0.717
40. Is able to recommend referral to a sexual health clinic when necessary	0.286	0.379	0.588

**Table 2:** Correlation coefficients for the Questionnaire Assessing Gynecologic Oncology Nurses’ Knowledge, Attitude, and Practice in Providing Sexual Health Care.

Item	Knowledge	Attitude	Practice
Knowledge	1		
Attitude	0.562	1	
Practice	0.880	0.583	1
Square root of AVE	0.912	0.757	0.808

**Table 3:** The heterotrait–monotrait ratio (HTMT) of the Questionnaire Assessing Gynecologic Oncology Nurses’ Knowledge, Attitude, and Practice in Providing Sexual Health Care.

Item	Knowledge	Attitude	Practice
Knowledge	-		
Attitude	0.585	-	
Practice	0.835	0.612	-

## Data Availability

The data presented in this study are available from the corresponding author upon reasonable request to safeguard sensitive individual information and privacy.

## References

[1] Bray F., Laversanne M., Sung H., Ferlay J., Siegel R.L., Soerjomataram I., Jemal A. (2024). Global cancer statistics 2022: GLOBOCAN estimates of incidence and mortality worldwide for 36 cancers in 185 countries. CA Cancer J. Clin..

[2] Dahouri A., Hosseinzadeh M., Sahebihagh M.H. (2026). Prevalence of sexual dysfunction and narrative synthesis of associated factors in patients with different type of cancer: A systematic review and meta-analysis. BMC Cancer.

[3] Almutairi W.M., Alsharif F., Al-Zahrani A., Bin Afeef N., Alkeai A., Alfakeeh H., Alzahrani A., Katooa N.E., Kassem F.K., Faheem W.A. (2025). Perceived Quality-of-Life Importance Among Saudi Gynecologic Cancer Survivors: Latent Class Analysis. Curr. Oncol..

[4] Vasconcelos P., Carrito M.L., Quinta-Gomes A.L., Patrão A.L., Nóbrega C.A., Costa P.A., Nobre P.J. (2024). Associations between sexual health and well-being: A systematic review. Bull. World Health Organ..

[5] Peleg S.N., Luria M., Sartorius G., Tripodi F., Lew-Starowicz M., Both S., Maseroli E., Reisman Y., Corona G. (2025). Clinical practice guidelines: Sexual dysfunction in gynecological cancer patients. Sex. Med..

[6] Wahbeh L., Ammar K., Abutaha S., Lataifeh I., Jaradat I., Abu-Hijlih R., Mohamad I., Salah S., Abu-Shanab S.M.S., Al-Sayyed M. (2025). Addressing sexual health needs in gynecological cancer survivors: Insights from a tertiary cancer center in Jordan. Front. Oncol..

[7] Paulsen A., Vistad I., Fegran L. (2024). Gynecological cancer survivors’ experiences with sexual health communication in nurse-led follow-up consultations. Acta Obstet. Gynecol. Scand..

[8] Suvaal I., Hummel S.B., Mens J.M., Tuijnman-Raasveld C.C., Tsonaka R., Velema L.A., Westerveld H., Cnossen J.S., Snyers A., Jürgenliemk-Schulz I.M. (2024). Efficacy of a nurse-led sexual rehabilitation intervention for women with gynecologic cancers receiving radiotherapy: Results of a randomised trial. Br. J. Cancer.

[9] Matthew A.G., Incze T., Stragapede E., Guirguis S., Neil-Sztramko S.E., Elterman D.S. (2025). Implementation of a sexual health clinic in an oncology setting: Patient and provider perspectives. BMC Health Serv. Res..

[10] Bhinder J.K., Kennedy S.K.F., Al Aadah C.F., Al-Khaifi M. (2025). 2024 SOGC, 2024 NCCN, 2022 ESO-ESMO, and 2018 ASCO: A comparison of female cancer survivorship guidelines for the management of sexual health concerns. Support. Care Cancer.

[11] Alqaisi O., Subih M., Joseph K., Yu E., Tai P. (2025). Oncology Nurses’ Attitudes, Knowledge, and Practices in Providing Sexuality Care to Cancer Patients: A Scoping Review. Curr. Oncol..

[12] Zarei F., Dehghani A., Ratansiri A., Ghaffari M., Raina S.K., Halimi A., Rakhshanderou S., Isamel S.A., Amiri P., Aminafshar A. (2024). ChecKAP: A Checklist for Reporting a Knowledge, Attitude, and Practice (KAP) Study. Asian Pac. J. Cancer Prev..

[13] Papadopoulou C., Sime C., Rooney K., Kotronoulas G. (2019). Sexual Health Care Provision in Cancer Nursing Care: A Systematic Review on the State of Evidence and Deriving International Competencies Chart for Cancer Nurses. Int. J. Nurs. Stud..

[14] Jopson R., Appleyard R. (2026). Encouraging a Patient-Centred Approach to Education, Designing an eLearning Platform to Enhance Knowledge of HPV Positive Head and Neck Cancer: A Delphi Consensus Study. J. Cancer Educ..

[15] DeVellis R.F., Thorpe C.T. (2021). Scale Development: Theory and Applications.

[16] De Vet H.C., Terwee C.B., Mokkink L.B., Knol D.L. (2011). Measurement in Medicine: A Practical Guide.

[17] Polit D.F., Beck C.T., Owen S.V. (2007). Is the CVI an acceptable indicator of content validity? Appraisal and recommendations. Res. Nurs. Health.

[18] Brown T.A. (2015). Confirmatory Factor Analysis for Applied Research.

[19] Crowley S.A., Foley S.M., Wittmann D., Jagielski C.H., Dunn R.L., Clark P.M., Griggs J.J., Peterson C., Leonard M., An L.C. (2016). Sexual health concerns among cancer survivors: Testing a novel information-need measure among breast and prostate cancer patients. J. Cancer Educ..

[20] Winterling J., Lampic C., Wettergren L. (2020). Fex-Talk: A Short Educational Intervention Intended to Enhance Nurses’ Readiness to Discuss Fertility and Sexuality with Cancer Patients. J. Cancer Educ..

[21] Albers L.F., Bergsma F.B., Mekelenkamp H., Pelger R.C.M., Manten-Horst E., Elzevier H.W. (2022). Discussing Sexual Health with Adolescent and Young Adults with Cancer: A Qualitative Study Among Healthcare Providers. J. Cancer Educ..

[22] Krouwel E.M., Albers L.F., Nicolai M.P.J., Putter H., Osanto S., Pelger R.C.M., Elzevier H.W. (2020). Discussing Sexual Health in the Medical Oncologist’s Practice: Exploring Current Practice and Challenges. J. Cancer Educ..

[23] Walker L.M., Wiebe E., Turner J., Driga A., Andrews-Lepine E., Ayume A., Stephen J., Glaze S., Booker R., Doll C. (2021). The Oncology and Sexuality, Intimacy, and Survivorship Program Model: An Integrated, Multi-disciplinary Model of Sexual Health Care within Oncology. J. Cancer Educ..

[24] Chen W., Ma Q., Chen X., Wang C., Yang H., Zhang Y., Ye S. (2021). Attitudes and Behavior of Patients with Gynecologic Malignancy Towards Sexual Issues: A Single-institutional Survey. J. Cancer Educ..

[25] Mrad H., Chouinard A., Pichette R., Piché L., Bilodeau K. (2024). Feasibility and Impact of an Online Simulation Focusing on Nursing Communication About Sexual Health in Gynecologic Oncology. J. Cancer Educ..

[26] Bae E.J., Kim Y.H. (2024). Development of Integrated Supportive Care Nursing Competence Scale for Cancer Survivors. Healthcare.

[27] Reynolds K.E., Magnan M.A. (2005). Nursing attitudes and beliefs toward human sexuality: Collaborative research promoting evidence-based practice. Clin. Nurse Spec..

[28] Wilson M.E., Williams H.A. (1988). Oncology nurses’ attitude and behaviors related to sexuality of patients with cancer. Oncol. Nurs. Forum.

[29] Gamel C., Hengeveld M.W., Davis B., Van der Tweel I. (1995). Factors that influence the provision of sexual health care by Dutch cancer nurses. Int. J. Nurs. Stud..

